# Supplementary material for: Molecular Composition and Biological Activity of a Novel Acetonitrile–Water Extract of Lens Culinaris Medik in Murine Native Cells and Cell Lines Exposed to Different Chemotherapeutics Using Mass Spectrometry
Source: Cells. 2023 Feb 10;12(4):575. doi: 10.3390/cells12040575 (PMC9953783; doi:10.3390/cells12040575)
Supplement: Supplementary file 1 [file cells-12-00575-s001.zip › cells-2152314-supplementary.pdf]

APPENDIX FILE

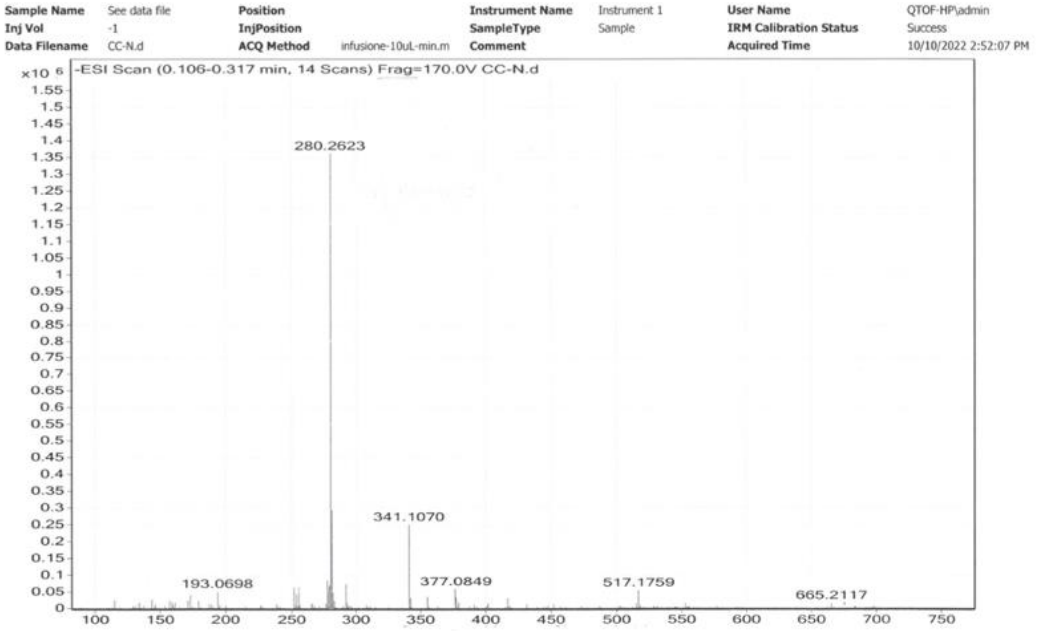

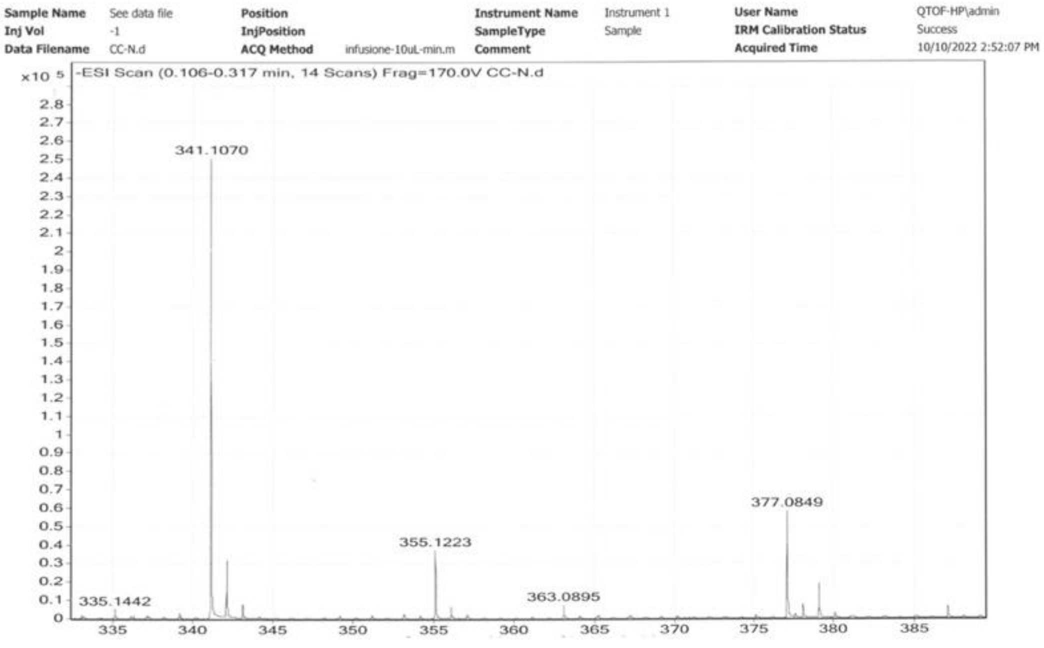

Figure S1. Liquid chromatography-mass spectrometry (LC-MS) of the acetonitrile-water extract 2E0217022196DIPFARMTDA. The first picture shows all the peaks obtained, of which the most abundant are more visible. The second picture shows only the interval in which there are more significant peaks.

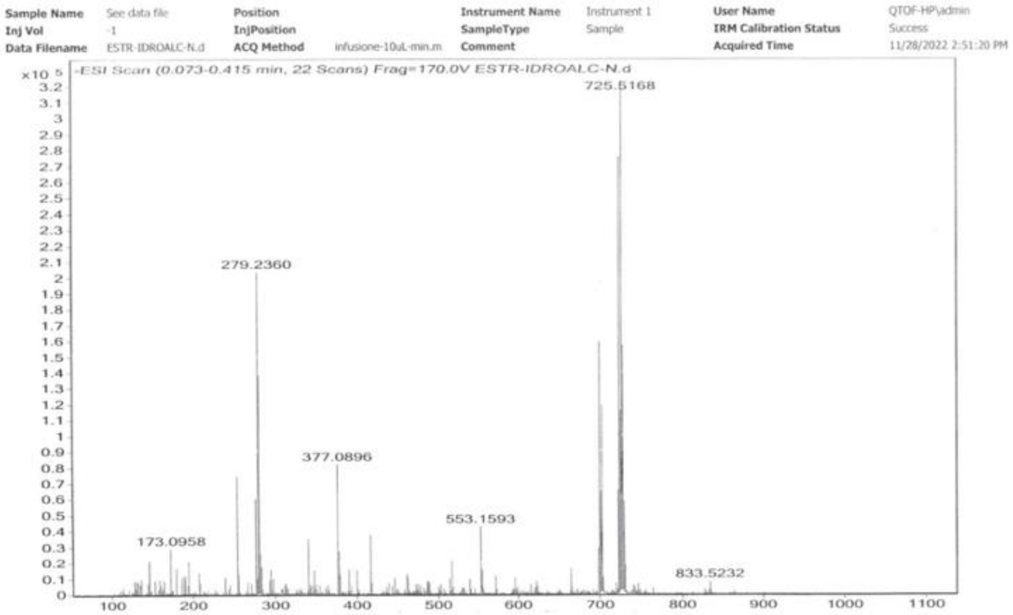

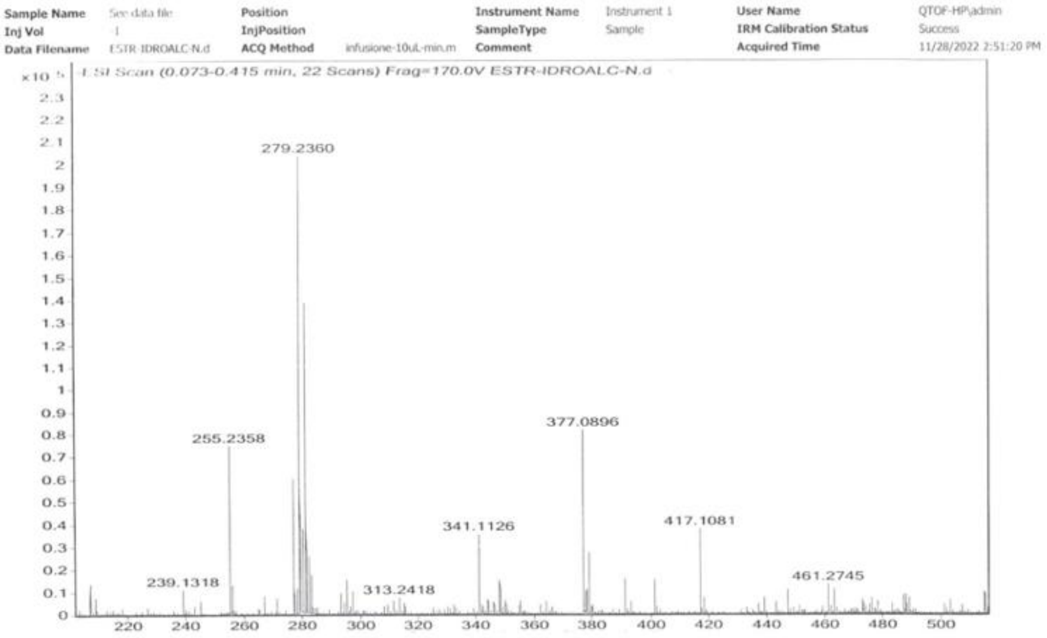

Figure S2. Liquid chromatography-mass spectrometry (LC-MS) of the hydroalcoholic extract 1E030521DIPFARMTDA. The first picture shows all the peaks obtained, of which the most abundant are more visible. The second picture shows only the interval in which there are more significant peaks mostly due to sugars.

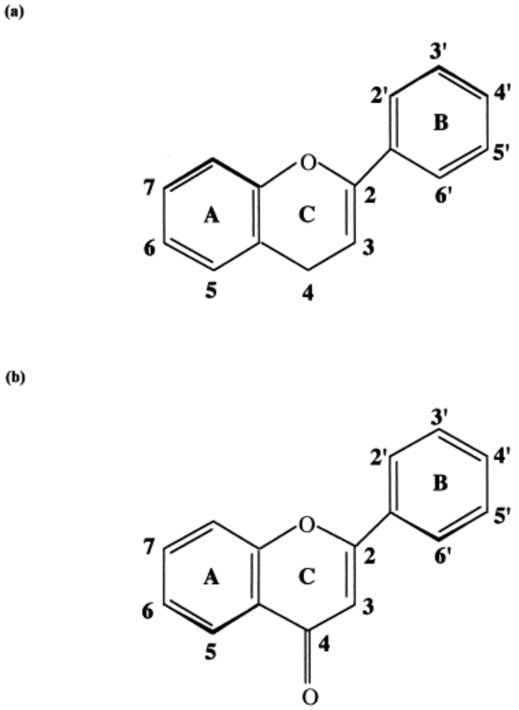

Figure S3. Flavan nucleus (a) and 4-oxo-flavonoid nucleus (b).

31150382	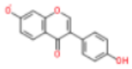	$C_{15}H_9O_4$	253.2301
24784790	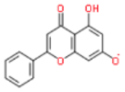	$C_{15}H_9O_4$	253.2301

Figure S4. Flavons found on Chemspider, are similar to apigenin.

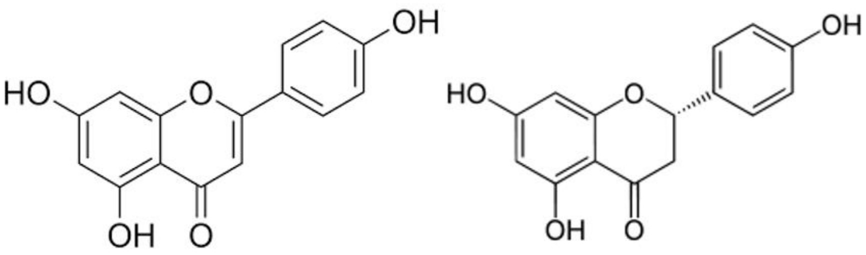

Figure S5. Molecular structure of Apigenin on the left and Naringenin on the right.

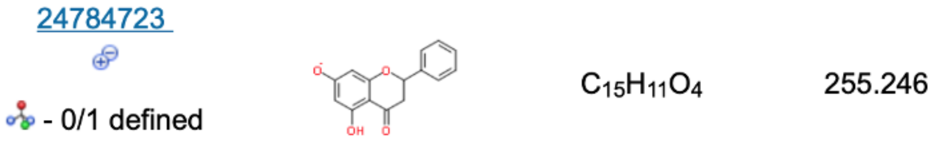

Figure S6. Flavanon is found on Chemspider, which is similar to naringenin.

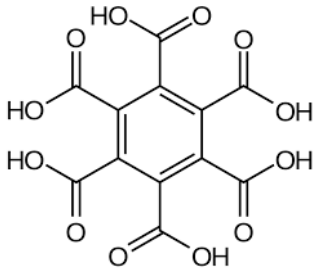

Figure S7. Chemical structure of mellitic acid.

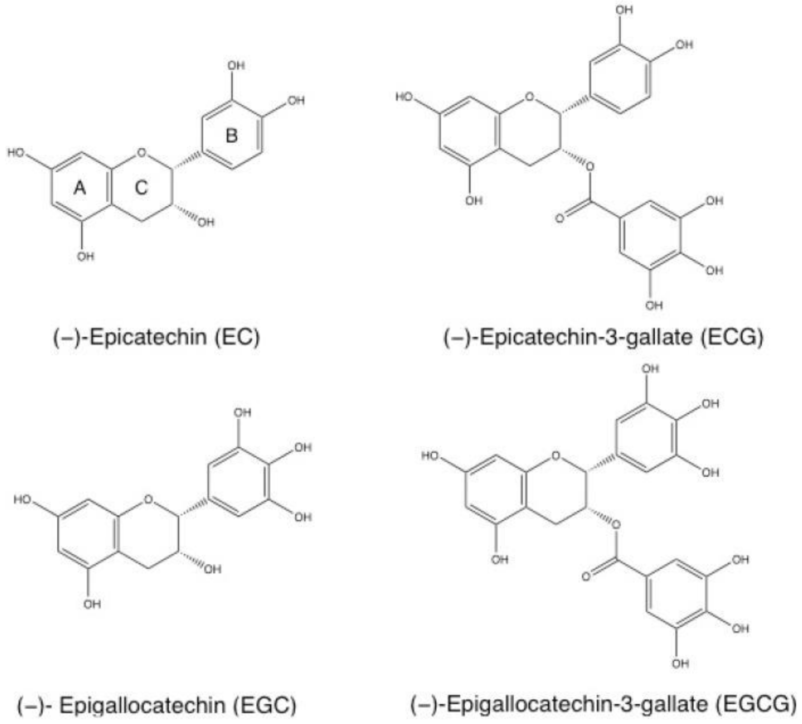

Figure S8. Chemical structure of (-) - epigallocatechin (EC), (-) - epigallocatechin-3-gallate (ECG), (-) - epigallocatechin (EGC) and (-) - epigallocatechin-3-gallate (EGCG) [15].

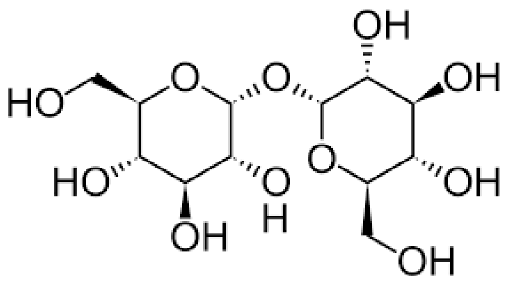

Figure S9. Chemical structure of Trehalose.

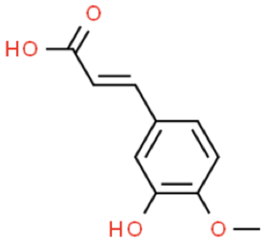

Figure S10. Chemical structure of 3-hydroxy-4-methoxycinnamic acid (isoferulic acid).

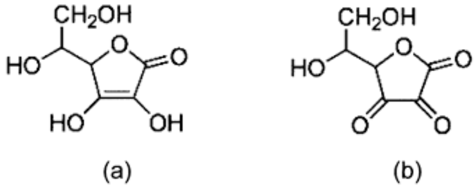

Figure S11. Structures of ascorbic acid(a) and dehydroascorbic acid(b).

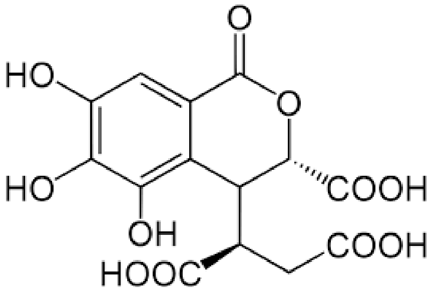

Figure S12. Chemical structure of chebulic acid.

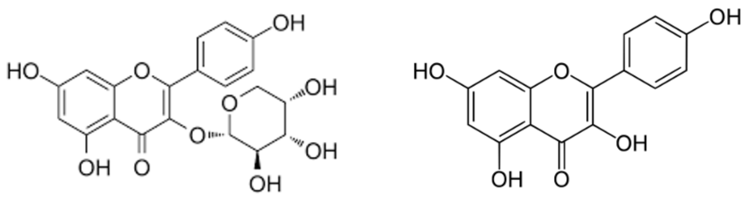

Figure S13. Chemical structure of kaempferol 3-O-arabinoside on the left and of kaempferol on the right.
